# Airway Management of a Patient With a Giant Frontoethmoidal Encephalocele: A Pediatric Case Report

**DOI:** 10.7759/cureus.49333

**Published:** 2023-11-24

**Authors:** Dulce Pereira, João Lusquinhos, Patrícia Santos

**Affiliations:** 1 Anaesthesiology, Centro Hospitalar Tondela-Viseu, Viseu, PRT; 2 Anaesthesiology, Centro Hospitalar Universitário de São João, Porto, PRT

**Keywords:** multidisciplinary approach, neuroanesthesia, airway management, difficult airway, meningoencephalocele

## Abstract

Meningoencephalocele is a rare congenital midline defect of cranial bone fusion characterized by herniation of the brain and meninges through the skull. In addition to the challenges of managing a major neurosurgical procedure in a pediatric patient, airway management in this group of patients requires advanced skills, and a difficult airway should be anticipated from the start. Since awake intubation is not an option in most pediatric cases with airway anatomy abnormalities and maintaining an adequate seal with a pediatric face mask is often impossible, airway management in patients with these lesions is highly challenging. We present the case of a 12-month-old girl with a postnatal diagnosis of frontoethmoidal meningoencephalocele who underwent craniotomy, followed by encephalocele resection, subsequent frontal cranioplasty, and reconstruction of the nasal bone defect. We discuss the timely adaptation of an adult face mask (size five) rotated 180º over the patient’s entire face to perform adequate preoxygenation and spontaneous ventilation assistance with hand-bag ventilation after the inhalational induction of general anesthesia. After obtaining adequate depth of anesthesia, an initial video laryngoscopy with pediatric Medan® was performed. The epiglottis and vocal cords were identified, and rocuronium was administered. After complete muscle relaxation, another video laryngoscopy was performed and orotracheal intubation was successful on the first attempt. As an alternative airway, we planned orotracheal intubation using a pediatric fiberoptic bronchoscope with the aid of a laryngeal mask airway if required. As a rescue measure, we also ensured that an otolaryngologist was present in the operating room if a tracheostomy was deemed necessary. We aim to raise awareness of the importance of safe practices in anesthesia, reinforce preventive measures during careful airway examination, and plan approach strategies.

## Introduction

Frontoethmoidal meningoencephalocele (FEM) is a congenital defect characterized by the herniation of the brain and meninges through a skull defect between the frontal and ethmoidal bones [1]. In untreated patients, an extruding mass of brain tissue and cerebrospinal fluid results in an expanding lesion and facial deformation. Additionally, it can erode the medial orbital walls or laterally displace the orbits. Surgical correction involves bicoronal incision, hernial sac removal, dural repair, and defect closure [2].

FEMs have a relatively high incidence in Southeast Asia (1:5000 live births) than in Western Europe, North America, and Australia [3,4]. The etiology of FEM involves a combination of genetic and environmental factors. An epidemiological study in Burma reported a higher prevalence among rural communities and rice farmers [5]. The mechanisms underlying congenital encephaloceles remain uncertain, although the involvement of defective anterior neural tube closure has been determined. Sincipital and basal varieties are thought to result from defective development of the prosencephalic neural crest tissue, as suggested by the patency of the midline foramina, which is open only transiently during normal craniofacial development [6].

In addition to the challenges of managing a major neurosurgical procedure in pediatric patients, airway management in this group of patients can require advanced skills and a difficult airway should be assumed and planned, particularly when considering difficult bag-mask ventilation and laryngoscopy.

This article was previously presented as a meeting abstract at the Euroanaesthesia 2022 on June 05, 2022.

## Case presentation

A 12-month-old girl, weighing 8.7 kg, with American Society of Anesthesiologists (ASA) physical status classification II, and with a postnatal diagnosis of a FEM, was scheduled for craniotomy, encephalocele resection, subsequent frontal cranioplasty, and nasal bone defect reconstruction. Surgical management involved a multidisciplinary approach, including anesthesiology, neurosurgery, ophthalmology, maxillofacial surgery, and otolaryngology. There was no history of previous anesthetic procedures except for sedation for magnetic resonance imaging (MRI), which was uneventful.

**Figure 1:**
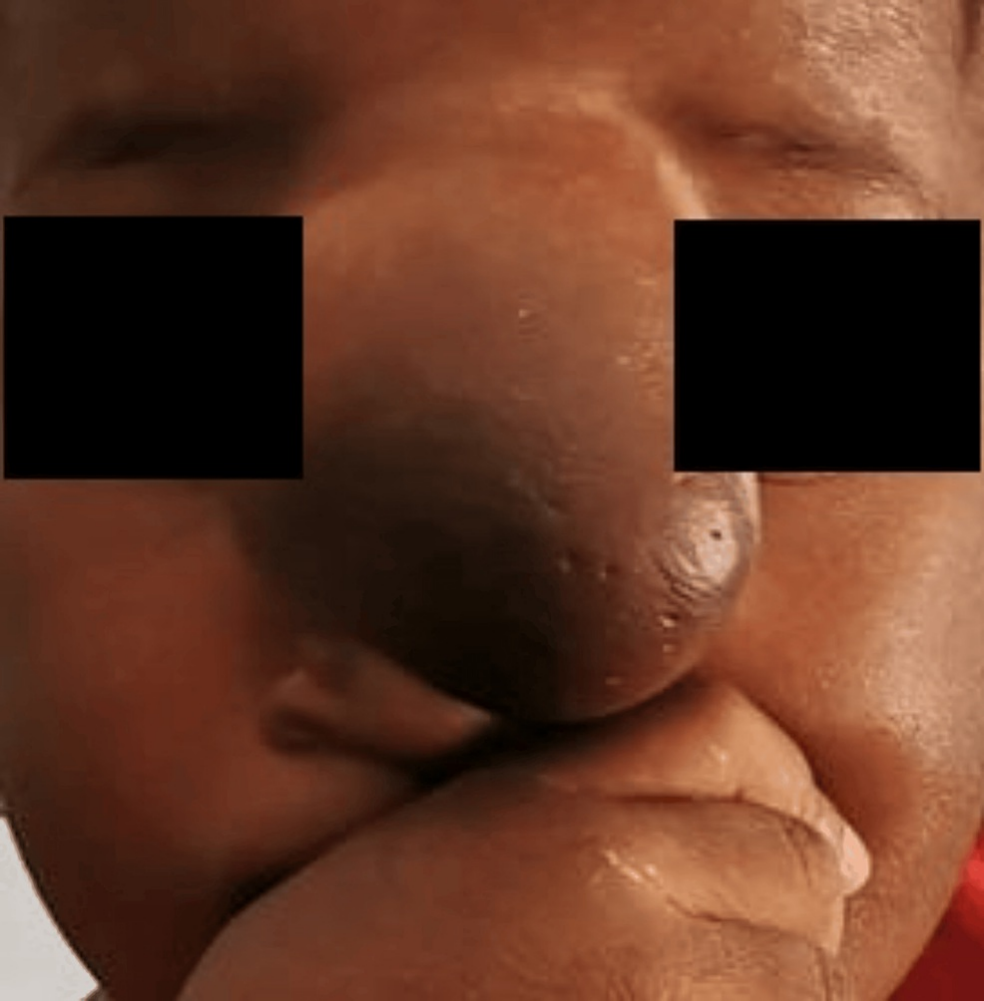
Frontoethmoidal encephalocele.

During the pre-anesthetic consultation, physical examination revealed a soft meningoencephalocele covered with intact skin extending from the patient’s glabella to the nasal alae, with a slight deviation to the left and partial occlusion of the field of vision (Figure 1), alongside an associated intermittent strabismus; therefore, difficult mask ventilation was immediately anticipated. No neurological deficits or other congenital abnormalities were observed, and the remaining findings were unremarkable. She had no relevant medical or allergic history.

**Figure 2:**
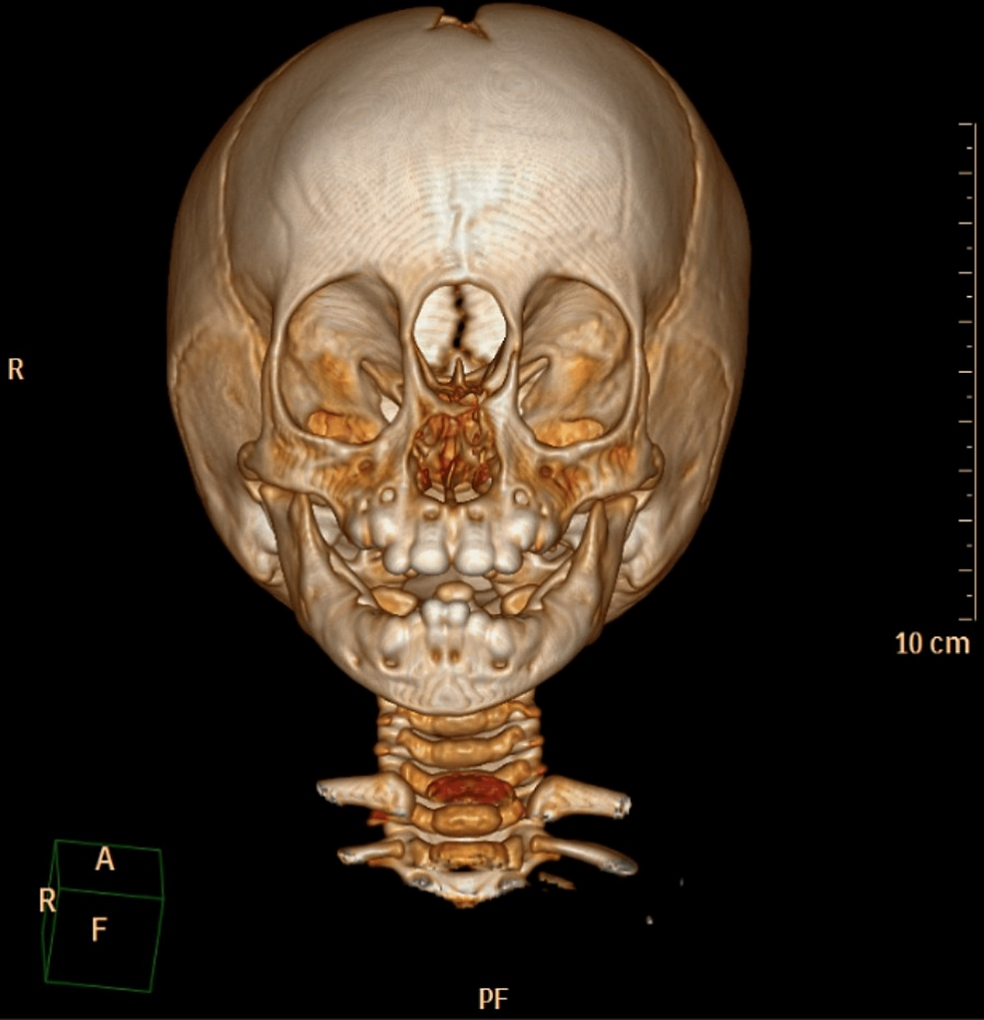
Three dimensional CT reconstruction showing extensive bone defect located at the anterior portion of the skull through which there was herniation of the meninges and brain parenchyma.

**Figure 3:**
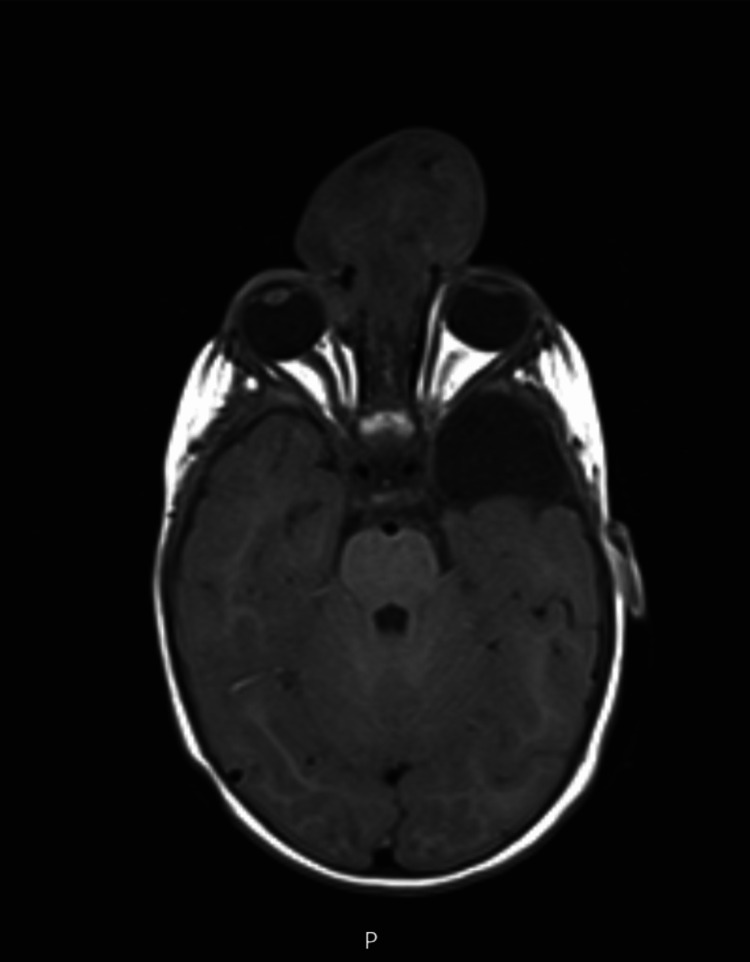
T1-weighted MRI of the frontoethmoidal encephalocele showing herniation of meninges and brain parenchyma through a bone defect and a meningoencephalic herniation into the right extraconical compartment.

Computed tomography (CT) and MRI of the brain revealed an extensive bone defect located at the anterior and medial portions of the base of the skull through which there was marked herniation of the meninges and brain parenchyma (Figures 2, 3).

The bone defect was accompanied by a depression of the cribriform plate, deformation of the anterior region of the nasal cavity, and bulging of the medial wall of the right orbit. There was also a solution for bone continuity through which there was a meningoencephalic herniation into the adjacent extraconical compartment. The herniated brain parenchyma had a dysplastic appearance, including cystic and necrotic areas, and static vessels. There has also been a reference to dysmorphia of the sulci and the presence of zones with dysplastic characteristics in the cortical parenchyma of the frontal lobes remaining inside the intracranial compartment. Additional preoperative laboratory results, including a full blood count, complete biochemical evaluation of renal function, and coagulation, showed no alterations.

As far as antiepileptic cover is concerned, since there is no suspected preoperative epileptic activity, prophylactic antiepileptic medication was not administered, as is usual in our institution. No premedication was given. The CT scan was also analyzed with the neurosurgical team, and it was considered that there was no reason for intracranial pressure monitoring or measures regarding to prevention of increased intracranial pressure since the meningoencephalocele would be removed.

**Figure 4:**
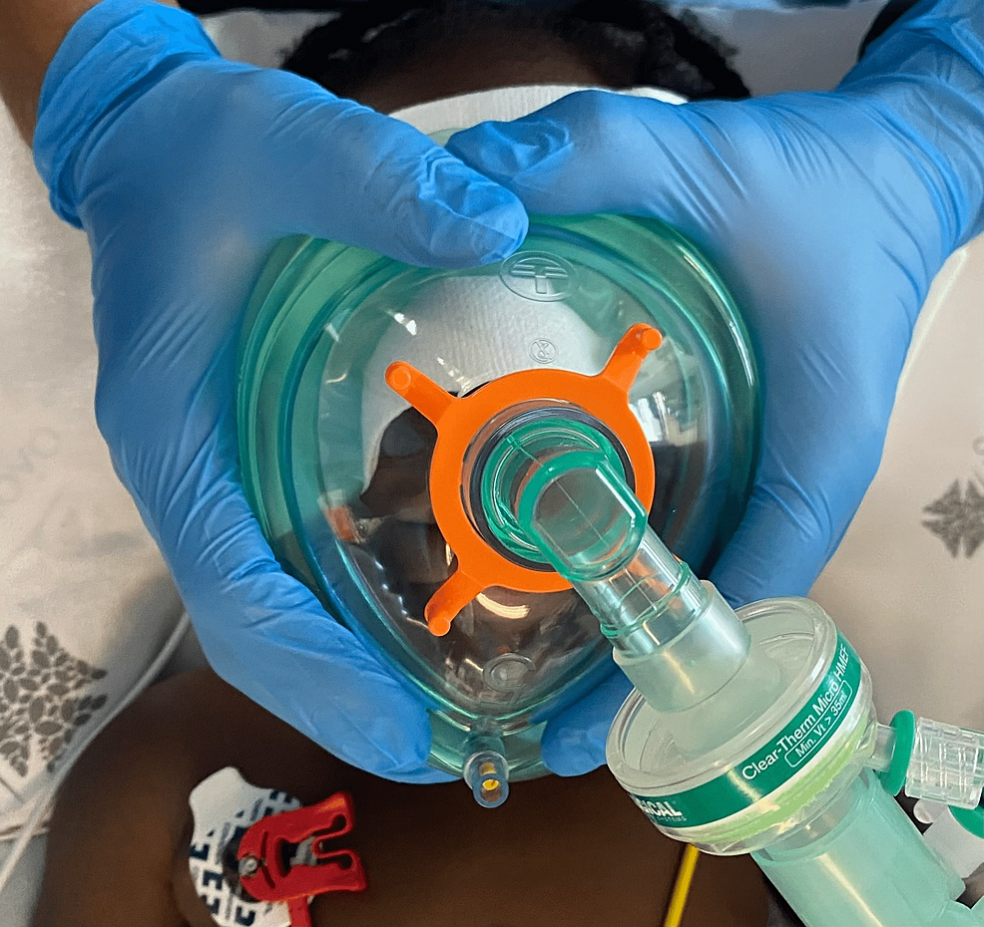
Adult face mask (size five) rotated 180º over the patient’s entire face.

In the operating room, the patient was placed in a supine position. Initial monitoring included basic standards for anesthetic monitoring from the American Society of Anesthesiologists, neuromuscular depth, Bispectral Index (BIS®), and Analgesia Nociception Index (ANI®). Temperature monitorization was achieved with an oesophageal probe. A warm blanket was used and because temperature was maintained between 36 to 36.5 degrees Celsius, no additional measures were needed. Since a pediatric face mask fit over the patient’s face was anticipated to be problematic, we placed an adult mask (size five with an adjustable air cushion) rotated 180º over the patient’s face to contour the skull and seal along the frontal bones, zygomatic bones, and mandible, incorporating the encephalocele (Figure 4).

A good seal was obtained using this modified technique, and adequate preoxygenation was possible. After denitrogenation, inhalation induction of anesthesia was performed with sevoflurane (1% - 8% v/v) in oxygen with the preservation of spontaneous ventilation. After obtaining adequate depth of anesthesia and confirmation of the possibility of assisting with and assuming hand-bag ventilation, an initial video laryngoscopy with pediatric Medan® was performed. The epiglottis and vocal cords were identified, and rocuronium was safely administered. After complete muscle relaxation, another video laryngoscopy was performed and orotracheal intubation was successful on the first attempt. As a viable alternative plan for the management of this predicted difficult airway, we prepared the material to proceed with orotracheal intubation using fiberoptic bronchoscopy (Ambu® aScope™ 4 Broncho Slim 3.8/1.2), with the aid of supraglottic airway devices if required. As a last rescue measure, it was ensured that an otolaryngologist was present in the operating room to prepare for a possible tracheostomy. An episode of mild bronchospasm immediately after intubation was promptly resolved using salbutamol, ipratropium bromide, 2 mg/kg ketamine, and hydrocortisone.

Anesthesia was subsequently maintained using a target-controlled infusion of propofol, paedfusor model, and remifentanil perfusion (0.05-1 µg/kg/min). After ensuring that the airway was secured, the ventilation parameters and depth of anesthesia were optimized, along with the verification of additional hemodynamic monitoring, which included invasive arterial blood pressure and pulse pressure variation achieved via femoral artery catheterization. A central venous line was placed in the opposite femoral vein, and a urinary catheter was inserted to monitor urine output.

No additional abnormal events, vasoactive drug administration, or blood component transfusion were observed. As far as blood losses are concerned, as expected, they were less than 5% of total blood volume. Simultaneously, the manutention fluids include a 6 mL/kg regarding insensitive losses. Hemodynamic stability was maintained during the seven-hour surgery. The procedure was successful, with complete resolution of the osseous malformation and excellent aesthetic results.

After the conclusion of the procedure, the residual neuromuscular blockade was reversed using sugammadex, the patient was extubated, and the anesthetic emergence was uneventful. The patient was subsequently admitted to the pediatric intensive care unit for further post-operative management and was discharged to the ward on the second postoperative day without any complications.

## Discussion

Congenital meningoencephaloceles are classified into the sincipital, basal, and occipital types. Sincipital varieties such as the one described in this case report can range from occult lesions to craniofacial deformities. Excluding cases of cerebrospinal fluid leakage, these can be treated electively, ideally early in infancy [7]. FEMs of this type appear to have a more favorable outcome than occipital or parietal FEMs, with an associated overall mortality of 7-20 % [8]. Most patients with this type of encephalocele have favorable developmental outcomes after surgery [6].

Although FEMs are rare congenital abnormalities, surgical repair of these lesions represents an anesthetic challenge. In addition to the inherently demanding management of major pediatric neurosurgeries, a difficult airway is anticipated, requiring careful and imaginative solutions. In this case report, a careful anesthetic plan was developed. Our initial airway approach included the application of an adult mask (size five) rotated 180º over the patient’s face to perform adequate preoxygenation [9,10], ventilation assistance, and hand-bag ventilation, always maintaining spontaneous ventilation, performing muscle relaxation, and subsequent intubation only after confirmation of visualization of the vocal cords through video laryngoscopy. Compression of the encephalocele was avoided because skin rupture can lead to brain exposure, hemorrhage, cerebral fluid leakage, or increased intracranial pressure. As an alternative, we also prepared for orotracheal intubation using fiberoptic bronchoscopy through a supraglottic device if required, maintaining spontaneous ventilation, as described above. High-flow nasal oxygen therapy or fiberoptic nasotracheal intubation was not an option, given the anatomical deformities, which included deformation of the anterior region of the nasal cavities and bulging of the medial wall of the right orbit. As a rescue measure, an otorhinolaryngologist was present in the operating room to prepare for the tracheostomy, if required. The anesthesiologist's ability to adapt and reinvent techniques, as demonstrated in this report, proved useful in handling this patient.

In addition to the concerns related to the airway approach, careful preoperative preparation and detailed intraoperative management are mandatory because pediatric patients represent a symbiosis of challenges related to their development and maturation of neurological and physiological systems. The main targets related to anesthesia management include the prevention of intracranial pressure increases, correct positioning to account for the long duration of the procedure, overlaying of surgical drapes over the child’s entire body, proper padding of pressure points, protection of the eyes, and easy access to intravenous lines and breathing circuits [11]. Maintaining normovolemia and consequent hemodynamic stability is a fundamental aspect, as is normothermia, as pediatric patients present a particularly high risk of perioperative hypothermia due to increased heat loss from the head, a high surface area-to-body mass ratio, and less effective thermoregulatory mechanisms [12]. Follow-up care in a pediatric intensive care unit is required for close ventilatory and hemodynamic monitoring and early detection of possible postoperative complications, such as seizures and infection [11].

## Conclusions

The inherent anesthetic implications of major neurosurgical interventions in pediatric patients involve proper positioning, hemodynamic monitoring, normothermia, and normovolemia maintenance. Meningoencephaloceles presents an additional challenge to airway management. An adult face mask may be useful for airway approach in patients with nasoethmoidal meningoencephalocele because it contours the patient’s face and seals the outer edges of the face, enabling preoxygenation, assistance, and hand-bag ventilation.

Regardless of the success of our first airway approach plan, alternatives, and rescue options must always be outlined before any intervention. Careful perioperative planning and collaboration between the surgical team and neuropediatric anesthesiologist are determining factors to ensure a successful outcome.

## References

[1] David DJ, Sheffield L, Simpson D, White J (1984). Fronto-ethmoidal meningoencephalocoele: morphology and treatment. Br J Plast Surg.

[2] Leelanukrom R, Wacharasint P, Kaewanuchit A (2007). Perioperative management for surgical correction of frontoethmoidal encephalomeningocele in children: a review of 102 cases. Paediatr Anaesth.

[3] Richards CG (1992). Frontoethmoidal meningoencephalocele: a common and severe congenital abnormality in South East Asia. Arch Dis Child.

[4] Suwanwela C (1972). Geographical distribution of fronto-ethmoidal encephalomeningocele. Br J Prev Soc Med.

[5] Aung Thu, Hta Kyu (1984). Epidemiology of frontoethmoidal encephalomeningocoele in Burma. J Epidemiol Community Health.

[6] Francis-West PH, Robson L, Evans DJ (2003). Craniofacial development: the tissue and molecular interactions that control development of the head. Adv Anat Embryol Cell Biol.

[7] Jimenez DF, Barone CM (2008). 15 encephaloceles, meningoceles, and dermal sinuses. Principles and Practice of Pediatric Neurosurgery.

[8] Macfarlane R, Rutka JT, Armstrong D (1995). Encephaloceles of the anterior cranial fossa. Pediatr Neurosurg.

[9] Krishna HM, Kundu R (2012). Adult face mask for inhalational induction in a child with maxillofacial injury. Anesth Essays Res.

[10] Thomas JJ, Ciarallo C (2015). Images in anesthesiology: facemask ventilation with a frontonasal encephalocele. Anesthesiology.

[11] Rath GP, Dash HH (2012). Anaesthesia for neurosurgical procedures in paediatric patients. Indian J Anaesth.

[12] Mutchnick I, Thatikunta M, Braun J (2020). Protocol-driven prevention of perioperative hypothermia in the pediatric neurosurgical population. J Neurosurg Pediatr.

